# Traits of *Heracleum sosnowskyi* Plants in Monostand on Invaded Area

**DOI:** 10.1371/journal.pone.0142833

**Published:** 2015-11-13

**Authors:** Igor V. Dalke, Ivan F. Chadin, Ilya G. Zakhozhiy, Ruslan V. Malyshev, Svetlana P. Maslova, Galina N. Tabalenkova, Tamara K. Golovko

**Affiliations:** Institute of Biology of Komi Scientific Centre of the Ural Branch of the Russian Academy of Sciences, Komi Republic, Russia; United States Department of Agriculture, UNITED STATES

## Abstract

The ability of giant hogweeds to form monodominant communities and even pure monostands in invaded areas has been well documented. Understanding of the mechanisms leading to monostand formation can aid in determining the limitations of existing community ecology models and establishing an effective management plan for invasive species elimination. The aim of this observational study was to investigate traits of *Heracleum sosnowskyi* plants (demography, canopy structure, morphology and physiology) of the plants in a pure stand in an invaded area useful for understanding potential monostand formation mechanisms. All measurements were performed in one typical *Heracleum sosnowskyi* monostand located in an abandoned agriculture field located in Syktyvkar city suburb (North-east Russia). This monostand consisted of five main plant growth stages: seed, seedling, juvenile, vegetative adult, and generative adult. Plants of all stages began to grow simultaneously shortly after the snowmelt, at the same time as spring ephemeral plant species grew. The density of generative plants did not change during the vegetation period, but the density of the other plant stages rapidly decreased after the formation of a tall (up to 2–2.5 m) and dense (Leaf area index up to 6.5) canopy. The canopy captured approximately 97% of the light. *H*. *sosnowskyi* showed high (several orders of magnitude higher than average taiga zone grasses) photosynthetic water use efficiency (6–7 μM CO_2_/μM H_2_O). Formation of *H*. *sosnowskyi* monostands occurs primarily in disturbed areas with relatively rich and well-moistened soils. Early commencement of growth, rapid formation of a dense canopy, high efficiency of light and water use during photosynthesis, ability of young plants to survive in low light conditions, rapid recovery of above-ground plant parts after damage, and the high density of the soil seed bank are the most important traits of *H*. *sosnowskyi* plants for monostand formation in invaded areas.

## Introduction

Invasion biology can be regarded as an interdisciplinary field of biological science. The problem of the dispersal of invasive species in a new environment is applicable to the fields of biogeography, community ecology and population biology. Invasive species provide a unique opportunity to study extreme cases of species distribution and ecosystem transformation. One such extreme case is *Heracleum sosnowskyi* Manden. (Apiaceae). *H*. *sosnowskyi* is one of a plant species that are determined to be invasive species [1], despite differing interpretations of the term “invasive species” [2–3]. Its wide distribution outside the limits of its natural range demonstrates successful passing of all four biological invasion stages [4]: transport, introduction, establishment, and spread. The ability of *H*. *sosnowskyi* and of the phylogenetically close *H*. *mantegazzianum* to form monodominant communities and even pure monostands within invaded areas has been well documented [5–7]. Thus, successful establishment of this species can be determined as pure stand emergence. The understanding of the mechanisms leading to monostand formation will determine the limitations of existing community ecology models [8–9] and aid in producing an effective management plan for this invasive species.

The aim of this observational study was to investigate traits of *Heracleum sosnowskyi* plants (demography, canopy structure, morphology and physiology) of the plants in a pure stand in an invaded area useful for understanding potential monostand formation mechanisms. These invaded areas are situated more than 2000 km north of the species’ natural range, in the Komi Republic (European North-east Russia). The biology of *H*. *sosnowskyi* as a prospective forage crop was studied in detail in the USSR in the second half of the 20th century [10]. European authors were the first to study *H*. *sosnowskyi* (along with *H*. *mantegazzianum)* autecology from an invasion biology point of view at the beginning of the 21st century [11–13]. However, important autecology and population traits of *H*. *sosnowskyi*, such as the rate and efficiency of photosynthesis, nitrogen and water use, leaf-area index, light consumption, population age structure, and dynamics, are still not available in the literature. Data on the ecophysiological traits of the native flora species have been collected in our laboratory over several years [14–20]. This allowed us to investigate specific traits of *H*. *sosnowskyi* that could explain the establishment success of the species in the invaded area.

## Materials and Methods

### Species studied

*Heracleum sosnowskyi* Manden is a perennial (biennial) monocarpic herbaceous plant that bears fruit once, and in 98–99% cases, dies after setting seeds. In North-east Russia, it can reach up to 3 m in height. The stem is hollow, and reaches up to 10 cm in diameter. Upper leaves do not have petioles, and have undivided three-blade laminae. Stem leaves are compound leaves with long petioles and three to five divided leaflets. Leaf blades are 30–150 cm long and 30–120 cm wide. Umbels are numerous (usually 5–6, up to 10); the central umbel is largest (up to 40 cm in diameter), with 30–75 peduncles, each with 18–60 pedicels. The flowering period occurs from July to August in the study area. *H*. *sosnowskyi* fruits are cremocarps, and consist of two mericarp seeds. The caudex is thick and short. The root system consists of a branchy taproot. The shoot of the adult generative *H*. *sosnowskyi* can be divided into three zones. Two zones are located underground: the vegetative shortened zone I, and the potential generative shortened zone II. The elongated generative zone III is located above ground. Juvenile and adult vegetative *H*. *sosnowskyi* plants contain zone I and zone II only. The above ground portion of the vegetative plant consists of leaves with long petioles [21]. The native range of *H*. *sosnowskyi* is located in the Caucasus region [10, 22]. In North-East Russia, the species grows mostly in disturbed areas: abandoned agricultural lands, waysides, and waste areas within inhabited localities.

### Study site. Micro-climate and agrochemical measurements

The majority of the data for this study was obtained in an *H*. *sosnowskyi* monostand population in an abandoned agriculture field situated in the Syktyvkar city (N 61.645556°, E 50.758611°). This land is owned by Komi Republic Public Institution Orphanage N 3 of Syktyvkar city. Written permission to publish the data presented in this paper has been given by Z. A. Vakhnina, director of the orphanage. This population was regarded as typical *H*. *sosnowskyi* monostand: cover (near 100%), median density of generative plants (1 plant per m^2^) and average height of generative plants (2.0–2.5 m). These values were similar to those values described in the literature [5–7] and were repeatedly observed at more than 10 sites in Syktyvkar city between 2008 and 2013.

The sum of growing day degrees was calculated according to method 1 given in [23].

Measurements of microclimate conditions were collected using data loggers LI-1400 (Licor Inc., USA), pyranometers, quantum and temperature sensors LI-190, LI-200 series (Licor Inc., USA), and a UV-radiometer TKA-PKM (TKA Scientific Instruments, Russia).

The soil of the study site was classified as agrozem in accordance with Classification and Diagnostics of Russian Soils [24], and this corresponds with the Anthrosols Reference Soil Group in World Reference Base for Soil Resources [25].

Soil samples were taken at 0–0.25 m depth. The soil was dried, ground, and purified to separate plant parts and seeds. Chemical analysis was performed in the Ecoanalit laboratory of the Institute of Biology of the Komi Scientific Center of the Urals Branch of the Russian Academy of Sciences. Total nitrogen contents were measured using an EA 1100 analyzer (CE Instruments, Italy). The exchangeable cations were extracted by ammonium acetate (pH 7) and quantified using an ICP Spectro CIROS anatomic emission spectrophotometer. The pH of the soil was measured using a potentiometer with a glass electrode.

### Demography data collection

The *H*. *sosnowskyi* life cycle can be divided into six age stages. The age stage identification is based on the form of the root system and divisions of the lamina [10]: seed, seedling (elongated seed-lobe, taproot without branching), juvenile (leaf with a crenate margin, taproot without branching), immature (root system is slightly branched, leaves are simple with lobate margin), vegetative adult (root system apparently branched, leaves are pinnately compound), and generative plants (presence of inflorescence, root system apparently branched, leaves are pinnately compound). In the present study, we combined immature and vegetative adult stages into one age stage: adult vegetative plants.

The number of seeds per plant was measured by counting peduncles and pedicels in central and side umbels on 30 plants. Because every pedicel holds two pieces of fruit, each containing two seeds, the following equation was used to calculate the number of seeds per plant:

$$N_{seed}=\;2\left(N_{p}N_{pc}N_{l}N_{pl}N_{pcl}\right)$$ (1)

Where, *N*_*seed*_: number of seeds per plant; *N*_*p*_: number of peduncles in central umbel; *N*_*pc*_: mean number of pedicels per peduncle in central umbel; *N*_*l*_: mean number of lateral umbels per plant; *N*_*pl*_: number of pedicels per peduncle in lateral umbel; *N*_*pcl*_: mean number of pedicels per peduncle in lateral umbel. The mean number of pedicels was determined for five randomly selected peduncles on every umbel. The size of the aerial seed bank was measured in March 2014 by a direct count of the seeds on 30 plants.

The density of the soil seed bank was determined with 30 soil cores, 50 mm in diameter, with a depth of 150 mm. The cores were taken every 5 m along three transects of 50 m length. Each soil core was divided to three soil layers: 0–5 cm, 5–10 cm and 10–15 cm (depth from surface). Samples were placed in 2 × 2 mm mesh bags. Samples were then washed in tap water to remove soil particles. The mesh bags were dried at room temperature, and the number of seeds in each bag was counted. Seed mass was measured with an analytical balance with a readability of 0.1 mg. Length and width of the flat side of the seeds were measured with a Vernier scale with a resolution of 0.1 mm. Seed area was estimated using the area enclosed by ellipse with the assumptions that the major axis was equal to seed length, and the minor axis was equal to seed width.

The density of *H*. *sosnowskyi* plants was measured in 1 m^2^ square plots. Sampling were performed along a transect in the east-west direction every 5 m.

### Morphometric measurements

Morphometric measurements were made in the same population on 20.07.2012 (n = 30, phase of mass flowering) and 01.07.2013 (n = 18, phase of mass budding–beginning of flowering). These data were collected in order to demonstrate the range of *H*. *sosnowskyi* linear dimensions. All measurements and sampling were performed along a transect in the east-west direction. Plants were sampled along the transect every 5 m. The following morphometric traits were measured: plant height, number and length of metameres, number of renewal buds per plant, number and area of leaves, and fresh and dry mass of roots, stems, and leaves. The area of the leaves was calculated using LAMINA software [26], and leaf photos were taken with a fixed pixel/mm ratio. The specific leaf area (SLA) values were calculated for the lamina without leaf petiole mass. Leaf area index (LAI) was determined as the total one-sided area of leaf tissue per unit ground surface area. Two methods for LAI determination were used. The first method used direct measurement of the total one-sided area of leaf tissue for individual plants and then multiplication by the median number of plants per unit ground surface area. The second (indirect) method for LAI determination was performed according to the theory described in the manual for AccuPAR PAR/LAI Ceptometer [27]. The fraction of transmitted photosynthetically active radiation (ratio of PAR measured below the canopy to PAR above the canopy) was measured with quantum sensor LI-190 SA (Licor Inc., USA). The zenith angle was 42°, the ratio of the length of the horizontal to the vertical axis was taken at 1.7 and the fraction of beam radiation was 0.86.

### CO_2_ exchange, pigment content and leaf transpiration measurements

The rate of CO_2_ exchange and leaf transpiration rate were measured on intact plants with LCPro+ (ADC BioScientific Ltd., UK). Light-harvesting complex II chlorophyll *a* fluorescence was measured with the PAM-2100 Portable Chlorophyll Fluorometer (Walz, Germany). Leaf chlorophyll and carotenoid contents were determined by spectrophotometer UV-1700 PharmaSpec (Shimadzu, Japan) in acetone extracts at 662 nm (chlorophyll a), 644 nm (chlorophyll b) and 470 nm (total carotenoids). Chlorophyll portion in Light-harvesting complexes was calculated, assuming that total chlorophyll b was located in the Light-harvesting complexes and the chlorophyll a to chlorophyll b ratio in this complex was equal to 1.2 [28].

### Statistical analysis

Statistical analysis was performed with R [29] and Statistica 10 software program (Statsoft Inc., CШA). All samples were tested for normality with Shapiro-Wilk tests. The selection of statistical inference methods was based on the results of the test with the significance level at 0.05. The central tendency (mean, median), standard deviation, standard error of the mean (SEM) and interquartile range (IQR) were calculated depending on the results of the Shapiro-Wilk test. The Kolmogorov-Smirnov test used as nonparametric methods for comparing two samples in the case of none-normal distribution data. The effects of leaf position factors on SLA were analyzed using an analysis of variance (ANOVA). The Duncan's test was performed after ANOVA for multiple comparison of sets of means.

CO_2_-exchange of plants was described by the Michaelis-Menten model. The nonlinear least-squares estimation of the parameters Pmax, K, Rd was performed using experimental data. Radiation rate at which photosynthesis efficiency reaches its maximum (irradiation rate of adaptation–IRA) and photosynthesis rate at IRA (P_IRA_) were found as coordinates of tangent to a curve after derivative computation of fitted Michaelis-Menten model. The light compensation point (LCP) was determined as the light intensity at which the total CO_2_ exchange is equal to zero. The photosynthetic quantum yield (QY), or photosynthetic efficiency was calculated as the tangent of the slope ratio of the light curve at low light intensities. Both of these parameters were found using linear regression with data on photosynthesis rate at low light intensities. The point coordinates of a tangent drawn from the origin to light response curve can be interpreted as plant functional traits [30]: the radiation rate at which photosynthesis efficiency reaches its maximum (adaptation irradiance, μM PAR m^-2^ s^-1^), and the photosynthesis rate with maximum energy convergence efficiency (net photosynthesis rate at adaptation irradiance, μM CO_2_ m^-2^ s^-1^).

The row data of most measurements used in the present study are available at https://zenodo.org/record/30998.

## Results

### Environmental and microclimate conditions in study site

The area occupied by *H*. *sosnowskyi* was approximately 2.5 ha. The measurements and observations were carried out during the vegetation periods in 2012 and 2013. Weather conditions were compared to the data collected at the Syktyvkar airport weather station (5 km from study area). The data were provided from the website "Reliable Prognosis": http://rp5.ru (weather station WMO ID = 23804). In both years, the snow melted in the last week of April at the study area. Thus, the duration of the *H*. *sosnowskyi* growing season in the Syktyvkar region was taken from May 1 to September 15. The sum of growing day degrees with a base temperature of 5°C in the growing season, was 1237°C in 2012 and 1272°C in 2013. The amount of precipitation was 583 mm in 2012 and 153 mm in 2013 for the same dates.

The soil of the study site is moderately acidic (pH = 5.77 ± 0.10). The humus, nitrogen (N total: 0.26 ± 0.04%), phosphorus (P_2_O_5_: 1080 ± 160 mg/kg) and potassium (K2O: 167 ± 17 mg/kg) content in the soil of the studied site were relatively high (in comparison with the native soils of the Syktyvkar region). The total content of nitrogen in the leaves of *H*. *sosnowskyi* was 2.4% dry mass.

The *H*. *sosnowskyi* monostand was growing in an open habitat. The photosynthetically active radiation above the canopy was 1500 μM photons m^-2^s^-1^, and dropped by 97% under the canopy (Table 1).

**Table 1:** Light irradiation regime in a *H*. *sosnowskyi* monostand (July, 2012). VS: light of the visible spectrum, PAR: photosynthetically active radiation (400–700 nm waveband); UV: ultraviolet radiation; symbols “a”, “b”, “c” designate the same groups in table columns segregated by Duncan's new multiple range test with a significance level at 0.05.

Sensor position	VS, W/m^2^	PAR, μM/m^2^ c	UV, W/m^2^
Above canopy	703 ± 61^a^	1513 ± 89^a^	5.2 ± 1.0^a^
Open space point 20 m from the monostand border	729 ± 37^a^	1436 ± 127^a^	8.7 ± 1.8^b^
Under canopy	131 ± 22^b^	49 ± 6^b^	0.5 ± 0.1^c^

### Demography

In the study area, the *H*. *sosnowskyi* plants began to grow immediately after snowmelt (in the last week of April). The median value of seedling density was 500 plants/m^2^. In the budding growth stage the seedling density decreased by a factor of 5. By the end of the vegetation period, the median density of seedlings and juvenile plants was reduced by one order of magnitude. The density of adult vegetative plants increased by up to 2.4 times (13 plants/m^2^) from the beginning of the growing season to the budding growth stage and significantly decreased at the end of the vegetation period (Table 2). The density of plants in the generative age stage was approximately 1–2 individuals/m^2^, with a median value of 1 individual/m^2^. The proportion of generative plants was less than 1% of the entire *H*. *sosnowskyi* population.

**Table 2:** The dynamics of plant density at different age stages in a *H*. *sosnowskyi* monostand: mean number of individuals per m^2^. “*”: The density of dead plants from the previous growing season. The median of the sample is shown. IQR of the sample is shown in parentheses.

Plant age stage	Growth stage		
	Start of vegetation (April)	budding (June–July)	End of vegetation (September–October)
Seedlings	509 (1019)	85 (141)	37 (36)
Juvenile	5.0 (8)	8 (9)	3 (2)
Adult vegetative	–	3.5 (2)	3 (2)
Generative	1.0 (0.3)	1.0 (2)	1 (1)

The generative *H*. *sosnowskyi* plants formed one main umbel on top of generative shoots and 5–7 lateral umbels on the lateral shoots (Table 3). The mean number of seeds formed on the main umbel was 2.6 (in 2012) and 9 (in 2013) times higher than the number of seeds formed on the lateral umbels. Each generative *H*. *sosnowskyi* individual produced a mean of 20400 ± 3200 seeds in 2012 and 14700 ± 1600 in 2013. Given the median density of generative plants, one plant per m^2^, the seed yield was 15000–20000 seeds/m^2^. The *H*. *sosnowskyi* seed dispersal mechanism is a combination of gravity and wind. The seed usually falls within few meters from the mother plant. Most seeds germinated in the next growing season after the obligatory cold period (0–5°C), with stratification from 3 to 4 months. Seeds collected in autumn 2013 were stored for 126 days in a wet chamber at 4°C. The germination rate was 64%.

**Table 3:** Morthometric traits of generative *H*. *sosnowskyi* plants in budding and flowering growth stages. “–” designates “no data”. The mean and standard deviation of samples is shown.

Trait	Growth stage	
	Budding (2013)	Flowering (2012)
Plant height, m	2.38 ± 0.25	2.97 ± 0.26
Metameres per aerial part of the shoot (the median ± semi-interquartile range)	4.9 ± 0.3	5.6 ± 0.7
Umbels number per plant	5.4 ± 2.3	7.1 ± 1.1
Total leaf area, m^2^	2.49 ± 0.85	1.38 ± 0.48
Taproot length, m	0.44 ± 0.17	–
Total fresh mass of the whole plant, g	5408 ± 1755	6265 ± 2250
Roots mass/above ground mass relation	0.15 ± 0.05	0.20 ± 0.07
Leaves mass/above ground mass relation	0.48 ± 0.11	0.29 ± 0.07

**Fig 1:**
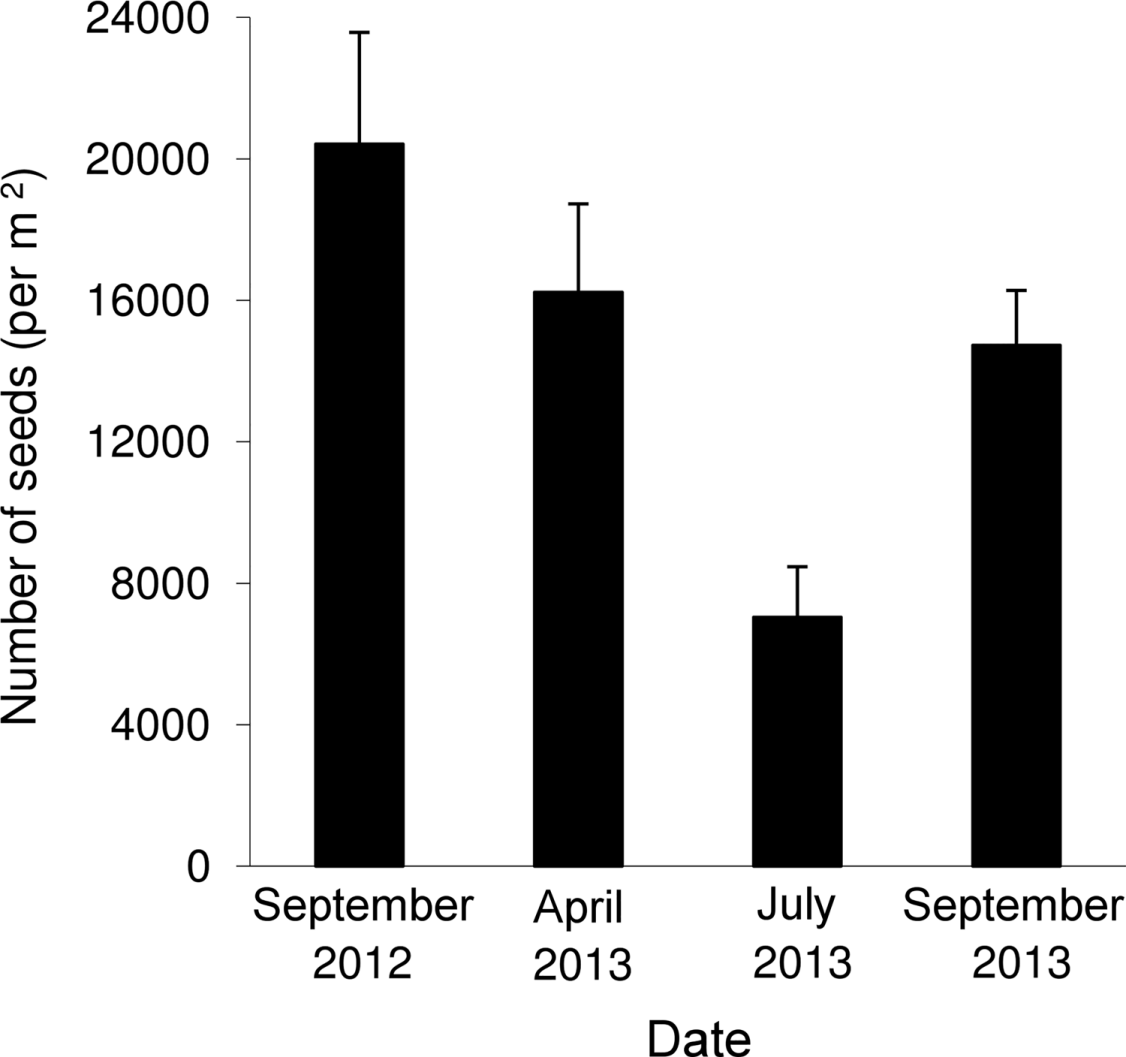
*Heracleum sosnowskyi* soil seed bank dynamics over a 12-month period (September 2012 to September 2013).

The soil seed bank contained up to 20000 seeds/m^2^ in the upper 15 cm of soil by the end of the growing season (Fig 1). The distribution of seeds in the vertical direction was heterogeneous: 80% of the seeds were found between 0 and 5 cm, 19% of seeds in the next layer (5–10 cm) and no more than 2% of seeds were at a depth of 10–15 cm. The mass of one air-dried seed was 1.7–20.6 mg (with a median of 13.0 mg). The area of the one flat side of the seed was 43–99 mm^2^ (with a median of 64 mm^2^). Accounting for the size of the soil seed-bank in the *H*. *sosnowskyi* monostand, there was about 1 m^2^ of seeds per 1 m^2^ of soil surface. The majority of the soil bank was replenished in autumn, after seed falling. The soil seed bank size did not change until April (Fig 1), when most part of the seeds had begun to germinate. Some of the seeds germinated below 1 m of snow cover, when the temperature of the frozen topsoil layer was -0.5°C. These seeds were able to develop well-formed seed lobes after being placed at room temperature.

A significant portion of the *H*. *sosnowskyi* seed yield remained in the umbels even after snow cover formation. Up to 80% of generative plants retained some portion of seeds in the air seed bank in March 2014. The number of these seeds was 0–1700 seeds/plant, approximately 8% ± 2% (n = 14) of the total seed yield per plant.

### Biomass and meristem allocation. Canopy structure

The *H*. *sosnowskyi* generative shoots reached a height of 2.5–3 m at the beginning of flowering (Table 3). The above ground part of the shoot consisted of an average of six metameres. The length of metameres ranged from 7–143 cm. The shortest metameres were found in the lower and upper part of the shoot. The length of the first three metameres was approximately 2/3 of the total shoot length (the median value was 2.1 ± 0.2 m). These three metameres bear the majority of the plant leaves, which comprise approximately 70–80% of the total leaf area. The leaf area per generative plant was 1.5–2.5 m^2^.

**Fig 2:**
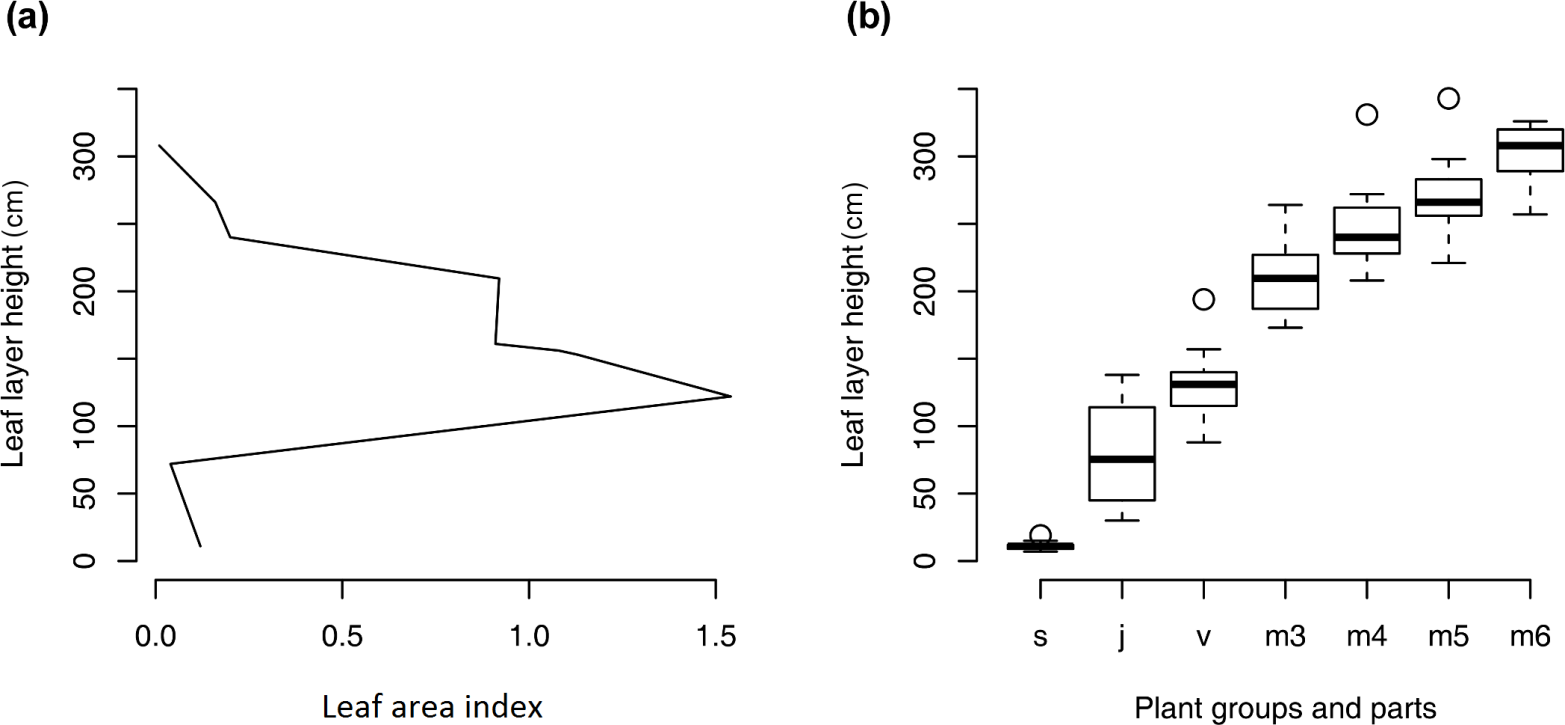
(a) Leaf area index and (b) height of *Heracleum sosnowskyi* leaf layers. Plant groups and plant parts: s–seedlings (N = 10); j—juvenile plants (N = 14), v—vegetative adult plants (N = 15); m3–m6 are generative plant metameres (N = 38): m3—leaves of first three metameres, and m4, m5, and m6—leaves of 4^th^, 5^th^, and 6^th^ metameres, respectively.

Four leaf layers can be determined in the *H*. *sosnowskyi* monostand canopy (Fig 2). Vertical distribution of total stand LAI showed two maximums: at the height of the second–third leaves of adult vegetative plants (120–150 cm) and at the height of the third leaves (metameres) of generative plants (200–230 cm). The total LAI of the *H*. *sosnowskyi* stand at the flowering stage was approximately 6.0. The LAI value determined by direct measuring of the leaf area and plant density was 6.1, and the value of LAI determined with the indirect method was 6.3.

**Fig 3:**
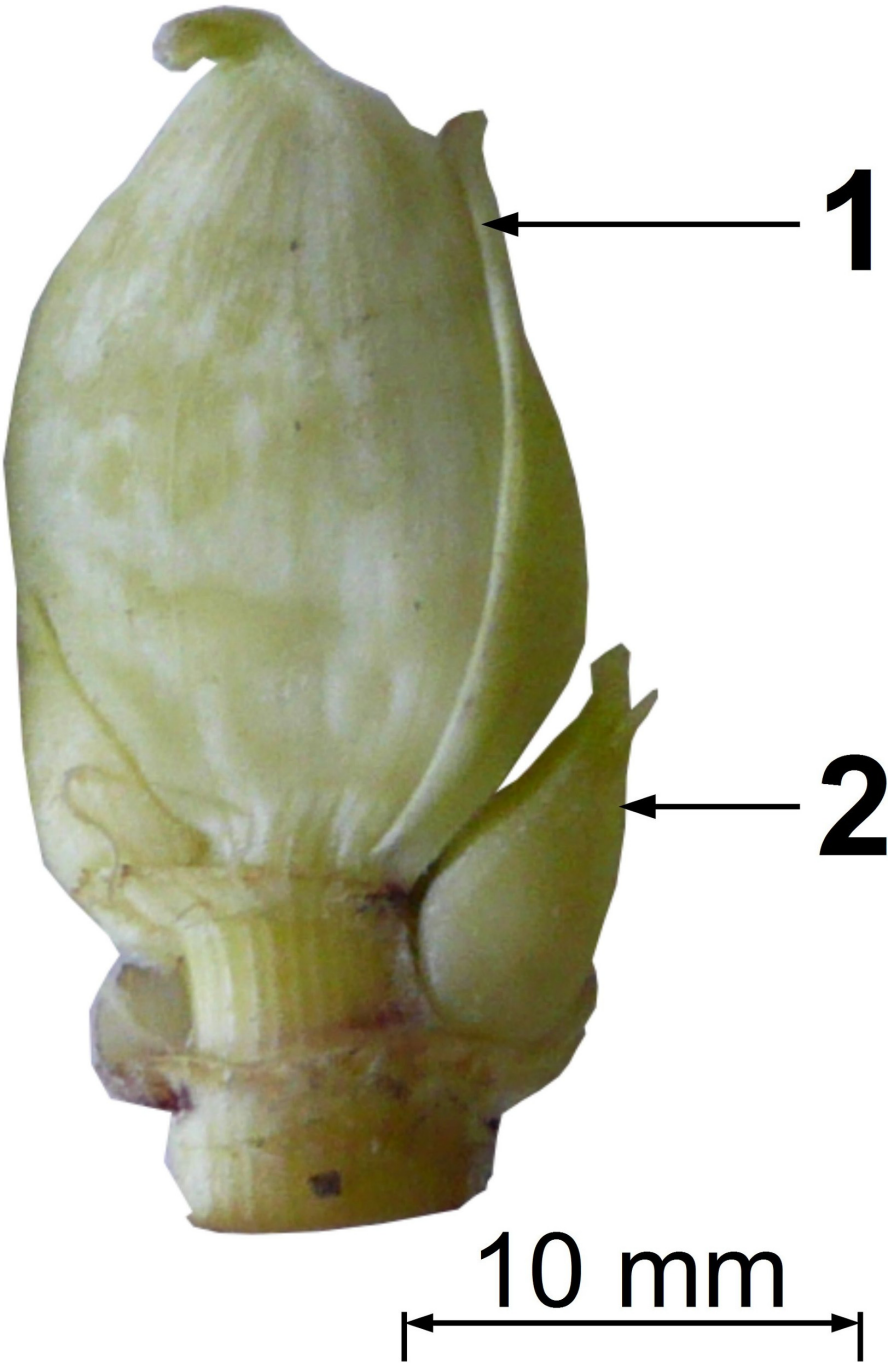
Dormant generative buds of adult vegetative *Heracleum sosnowskyi* plants. 1- terminal bud, 2- lateral bud.

**Fig 4:**
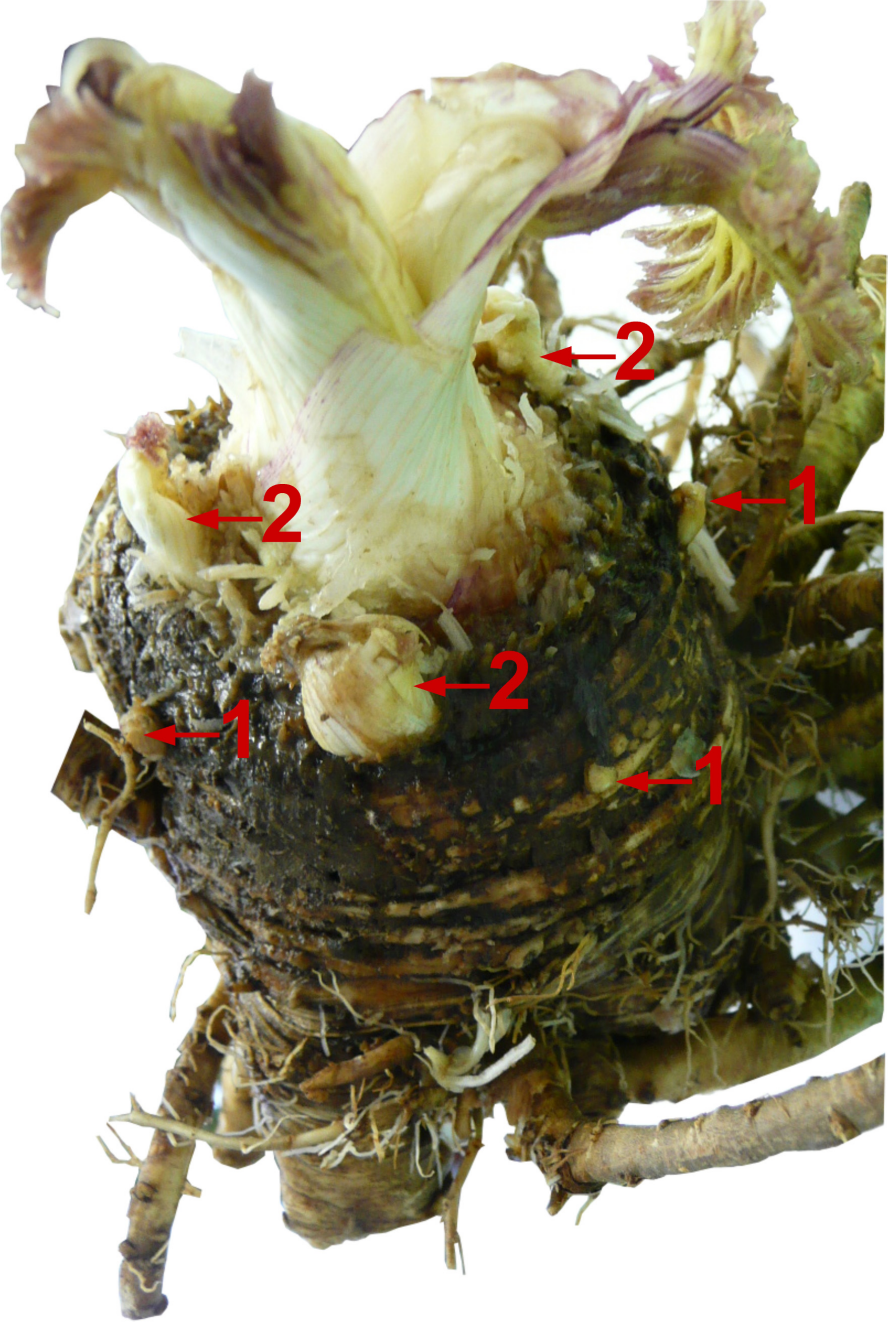
Generative *Heracleum sosnowskyi* plant underground portion. The caudex with roots and leaves (1- buds formed in zone II, 2- buds formed in zone I of shoot).

Between 5–6 dormant generative buds formed on zone II of generative plant shoots at the flowering growth stage. The mean length of the buds was 16 ± 4 mm, buried in the soil at the depth of 8–10 cm (Fig 3). At 2–3 years, the vegetative plants formed dormant buds in zone I and zone II (Fig 4) at the end of growing season. In rare cases (not more than 1–2%), the generative plants formed vegetative buds at the end of the growing season. Thus, a small portion of the *H*. *sosnowskyi* population are polycarpic. The total length of the caudex and taproot reached up to 200 cm; the caudex diameter was 8–13 cm.

**Fig 5:**
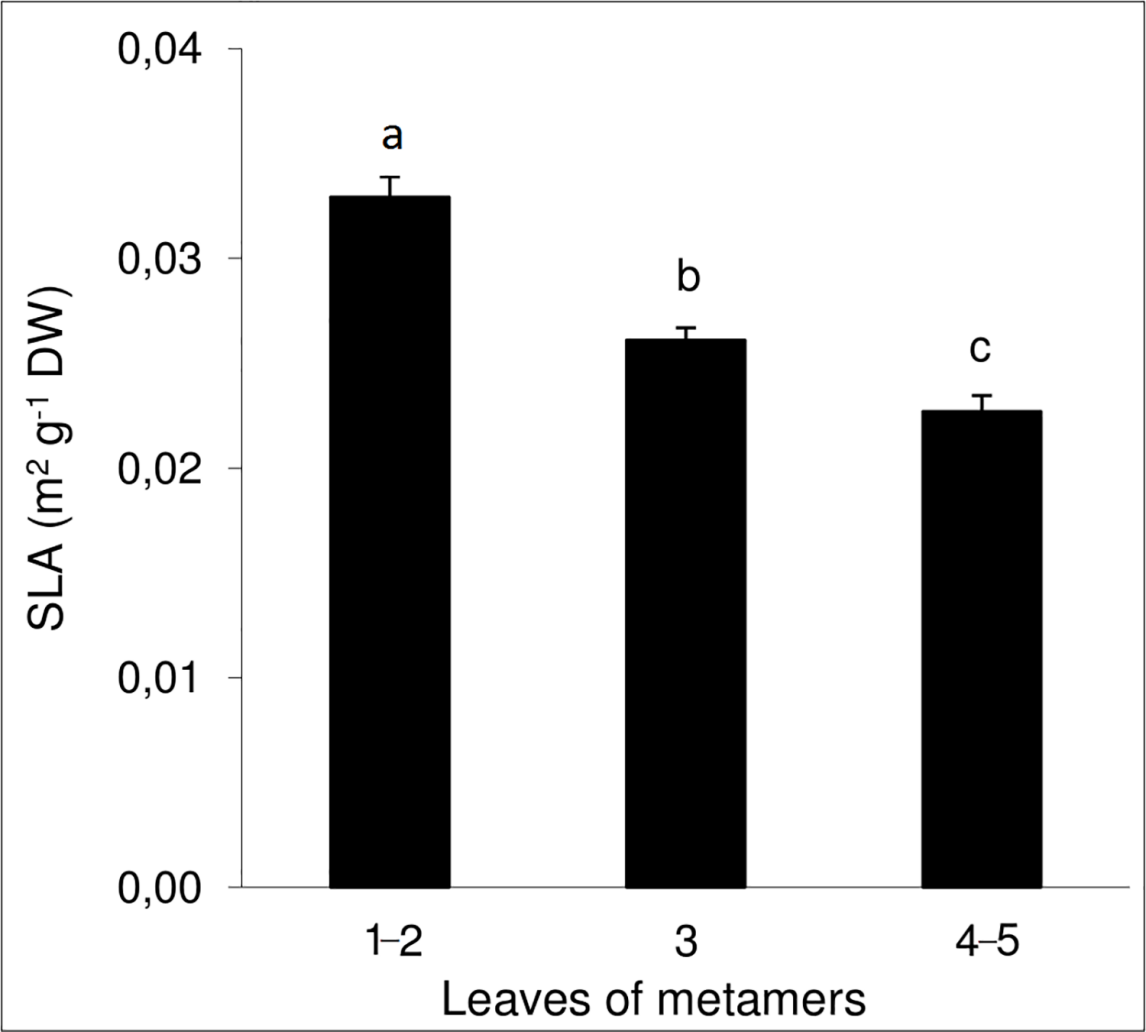
Specific leaf area (SLA) at different metameres stem of *Heracleum sosnowskyi*. Symbols “a”, “b”, “c” designate the same groups segregated by Duncan's new multiple range test with a significance level at 0.05.

The data on biomass allocation showed that *H*. *sosnowskyi* generative plants invested more than half of the assimilated carbon to supporting structures: stems and leaf petioles (Table 4). Umbels contribute up to 13% of the total biomass in the flowering stage. Despite the large size and high dry matter content, the portion of underground organs did not exceed 15% of the total plant dry weight (DW). *H*. *sosnowskyi* SLA without petiole ranged from 0.009–0.0125 m^2^/g DW. The SLA value depended on the leaf position on the shoot and was the lowest for leaves from the first two metameres (Fig 5).

**Table 4:** Biomass allocation of *H*. *sosnowskyi* generative plants in budding and flowering growth stages. “*”: difference is significant with *p* < 0.01 with a Kolmogorov-Smirnov test. The mean and standard deviation of samples is shown.

Plant part	Budding (2013)		Flowering (2012)	
	Fresh mass, g	Portion, %	Fresh mass, g	Portion, %
Leaf lamina	1039 ± 448	19 ± 4*	640 ± 252	10 ± 3*
Leaf petiole	1300 ± 849	23 ± 10*	938 ± 462	15 ± 5*
Stem	2193 ± 560*	42 ± 8	2851 ± 1141*	45 ± 4
Umbels	185 ± 83*	4 ± 2*	845 ± 295*	14 ± 3*
Caudex and roots	691 ± 311	13 ± 4	991 ± 396	16 ± 5
Whole plant	5408 ± 1755	100	6265 ± 2250	100

### Rate and efficiency of CO2 assimilation and water transpiration

As indicated above, the laminae of the first three metameres of *H*. *sosnowskyi* leaves were located approximately on the same level. Thus, the physiological and biochemical traits of first three metameres were statistically identical. The leaves of the first two metameres accumulated more chlorophyll than the upper leaves of the fourth and fifth metameres (Table 5). The Chlorophyll portion in Light-harvesting complexes changed from 56% in the lower leaves to 50% in the upper leaves (Table 5). Leaves of the upper and middle layers had a lower chlorophyll/carotenoid ratio because of lower chlorophyll content. The carotenoid content was the same for leaves of different metameres.

**Table 5:** Photosynthetic pigment content and their ratio in *H*. *sosnowskyi* leaves of different layers (flowering stage, June 2013). “Chl”: chlorophyll, “Car”: carotenoids. LHC-Chl–Chlorophyll portion in Light-harvesting complexes. Symbols “a”, “b” designate the same groups in table columns segregated by Duncan's new multiple range test with a significance level at 0.05. The mean and standard error of mean of samples is shown.

Leaf layer	Chl (*a + b*), mg/g DW	Chl *a/b*	Carotenoid, mg/g DW	Chl/Car	LHC-Chl, %
Upper (4^th^–5^th^ metameres)	4.46 ± 0.59^a^	3.50 ± 0.05^a^	1.13 ± 0.12^a^	3.86 ± 0.11^a^	49 ± 1^a^
Middle (3^rd^ metamere)	6.17 ± 0.17^ab^	3.38 ± 0.05^a^	1.49 ± 0.05^a^	4.15 ± 0.03^a^	50 ± 1^a^
Lower (1^st^–2^nd^ metameres)	7.18 ± 0.21^b^	2.94 ± 0.06^b^	1.43 ± 0.03^a^	5.01 ± 0.06^b^	56 ± 1^b^

The photosynthesis rate of the different leaf layers within generative plants were measured by the quantum yield of Photosystem II (PS II) and speed of CO_2_ gas exchange. In the generative plants, leaves of different layers did not have significantly different potential photochemical activity. The maximum quantum yield was 0.82 for leaves of the upper, middle and lower layers.

**Fig 6:**
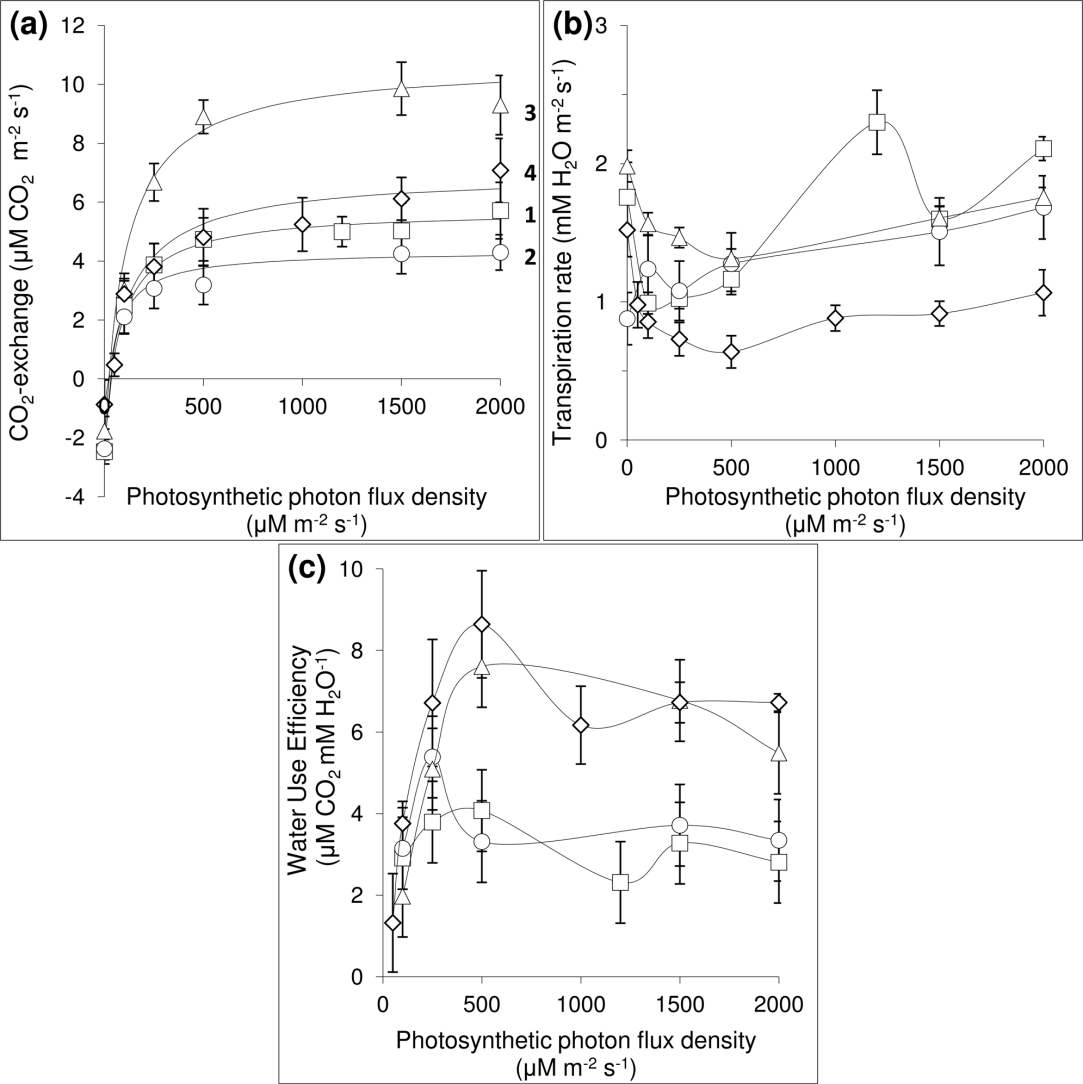
Net photosynthesis (a), transpiration rate (b) and water use efficiency (c) as a function of light in *Heracleum sosnowskyi* leaves. 1—seedlings, 2—juvenile plants, 3—vegetative adult plants, 4—generative plants.

Light use efficiency (LUE) of upper leaves was significantly higher than that of middle and lower layers. Thus, in low light levels (500 μM m^-2^ s^-1^ PAR ~ 25% of maximum PAR), a real quantum yield of PS II was approximately 80% of the maximum, and a quantum yield of PS II in lower and middle layer leaves was reduced by 30–40% (*p* = 0.023). The potential photosynthesis rate did not differ in leaves of different layers (Fig 6) in low light conditions (less than 25% of maximum PAR). The upper leaves showed a higher electron transport rate (ETR) then lower leaves did in light saturation point conditions (more than 1000 μM CO_2_ m^-2^ s^-1^ PAR) (Fig 6A).

As indicated above, the canopy of the *H*. *sosnowskyi* monostand consisted of four main leaf layers: seedlings, juvenile plants, adult vegetative plants and the first three metameres of generative plants. A detailed study of the CO_2_ exchange response to light conditions and transpiration rate of the leaves of this layer was performed (Fig 6). These leaves showed the following parameters of the Michaelis-Menten model for photosynthetic light response:

$$P=\frac{P_{max}PAR}{PAR+K}-Rd$$ (2)

*P* is CO_2_ exchange rate (net photosynthesis rate); *P*_*max*_ is maximum rate of photosynthesis; *PAR* is photosynthetic active radiation; *K*: the Michaelis-Menten constant (photosynthetic photon flux density at which *P* is one-half of *P*_*max*_); and *R*_*d*_: dark respiration. The values of parameters for *H*. *sosnowskyi* leaves of different age groups are presented in Table 6.

**Table 6:** The Parameters of the Modified Michaelis-Menten model. For all data presented, P < 0.05. The mean and standard error of mean of samples is shown.

Stages of Plant Growth	*P*_*max*,_ (μM CO_2_ m^-2^c^-1^)	*K*, (μM photons m^-2^c^-1^)	*Rd*, (μM CO_2_ m^-2^c^-1^)
Seedlings	8.02 ± 0.81	83 ± 29	2.28 ± 0.67
Juvenile Plants	6.70 ± 0.59	48 ± 21	2.36 ± 0.44
Vegetative Plants	12.70 ± 1.01	110 ± 30	1.97 ± 0.87
Generative Plants	7.90 ± 0.76	137 ± 52	0.94 ± 0.59

For the data presented P-value < 0.05.

The value of photosynthesis quantum yield in leaves of different layers ranged from 0.04–0.06 moles CO_2_/moles quanta of PAR. The mean value of the light compensation point (LCP) in leaves of seedlings and juvenile plants was up to 1.5 times higher than in the LCP in leaves of adult vegetative and generative plants (Table 7). Leaves of adult vegetative and generative plants achieved half the maximum photosynthesis rate at PAR 100–140 μM m^-2^ s^-1^. The leaves of seedlings and juvenile plants achieved the maximum photosynthesis rate at PAR 50–80 μM m^-2^ s^-1^ (Fig 6A).

**Table 7:** Parameters of the Light Curve of Net Photosynthesis in *Heracleum sosnowskyi* Leaves. QY: photosynthetic quantum yield (calculated as the tangent of the slope ratio of the light curve at low light intensities: 0–100 μM photons m^-2^c^-1^), LCP: Light Compensation Point is the light intensity at which the total CO_2_-exchange is equal to zero, P_MAX_: maximum photosynthesis rate, P_max_*—maximum photosynthesis rate calculated by SLA, IRA: intensity of radiation adaptation, P_IRA_: photosynthesis rate at IRA. For the QY P-value < 0.0001. The mean and standard error of mean of samples is shown.

Stages of Plant Growth	SLA, (m^2^ g^-1^ DW)	QY (μM photons μM CO_2_^-1^)	LCP, (μM photons m^-2^c^-1^)	P_max_, (μMCO_2_ m^-2^c^-1^)	P_max_*, (mgCO_2_ g DW^-1^h^-1^)	IRA_,_ (μM photons m^-2^c^-1^)	P_IRA,_ (μM CO_2_ m^-2^c^-1^)
Seedlings	0.063 ± 0.003	0.057 ± 0.011	43 ± 20	5.71 ± 0.96	56	95	2.00
Juvenile Plants	0.042 ± 0.002	0.051 ± 0.007	46 ± 14	4.29 ± 0.60	28	70	1.62
Vegetative Plants	0.016 ± 0.001	0.055 ± 0.009	32 ± 16	9.85 ± 0.90	25	71	3.03
Generative Plants	0.012 ± 0.001	0.043 ± 0.010	25 ± 18	7.08 ± 1.08	13	72	1.79

There was no difference in CO_2_ assimilation rates between different leaf groups over the range of PAR 0–100 μM m^-2^ s^-1^. The adult vegetative plants showed the highest CO_2_ assimilation rate when PAR was greater than 250 μM m^-2^ s^-1^. The leaves of the seedling, juvenile and generative plants assimilated CO_2_ 1.5–2 times slower than the leaves of the vegetative plants.

The ratio of CO_2_ assimilation rate to leaf DW showed an inverse pattern: CO_2_ uptake of the seedlings was approximately 60 mg CO_2_ g DW ^-1^ hour^-1^. This value was 2–4 times higher than CO_2_ uptake in leaves of the other plant age groups. This difference was due to a significant difference in SLA. The SLA of younger plant leaves was 1.5 to 5 times higher than leaves of other plant age groups (Table 7). The respiration rate made up to 35% of the CO_2_ assimilation rate in the leaves of young plants, and was two times higher than the leaves of older plants (Table 6).

The adaptation irradiance of leaves of all studied *H*. *sosnowskyi* was 70–100 μM PAR m^-2^ s^-1^ (Table 7). This value is similar to the light irradiation value below the *H*. *sosnowskyi* canopy, and an order of magnitude lower than the PAR reached in the *H*. *sosnowskyi* canopy on a sunny day (Table 1). The net photosynthesis rate at adaptation irradiance was approximately 1.5–2 μM CO_2_ m^-2^ s^-1^. This is 3–4 times lower than the photosynthesis rate at light saturation.

The rate of leaf transpiration ranged from 0.5–2.5 μM H_2_O m^-2^ s^-1^ under light conditions from 100–2000 μM m^-2^ s^-1^ PAR (Fig 6B). The maximum photosynthetic water-use efficiency was reached at a light level of 500 μM m^-2^ s^-1^ PAR. The photosynthetic water-use efficiency in leaves of different layers was relatively high: 60% of the maximum (Fig 6C). It can be estimated that 1 ha of an *H*. *sosnowskyi* monostand transpires approximately 3500–4500 kg of water and assimilates approximately 10–26 kg of carbon in 1 hour in full light condition.

## Discussion

Trait complex of *H*. *sosnowskyi* plants allow the species to establish disclimax monostands in invaded areas, which in turn allows this species to reach the next stage of the invasion process: dispersal. The relevant traits are early commencement of growth, rapid formation of a dense canopy, high efficiency of light and water use during photosynthesis, ability of young plants to survive in low light conditions, rapid recovery of above-ground plant parts after damage and a high density soil seed bank.

*H*. *sosnowskyi* plants begin to germinate from both seeds and underground shoot buds immediately after snowmelt, as early as spring ephemeral plant species do. A portion of the seeds germinate even before snowmelt, under snow cover at temperatures of -0.5°C. The plants form 100% monostand cover in the last week of May, earlier than most of the plants in the geographic region.

The *H*. *sosnowskyi* monostand consists of plants of all age stages: seed, seedling, juvenile, immature, vegetative adult, and generative plants. For the majority of the growing season, plants of all stages grow simultaneously. This leads to an *H*. *sosnowskyi* monostand LAI value (6.0–6.3) close to the theoretical optimum LAI for broad leaf ecosystems [31]. The canopy captures approximately 97% of the light. This allows *H*. *sosnowskyi* to capture most environmental resources (such as light) and prevent penetration of other plant species into the monostand. Multi-species community formation is possible if the species differ in their ability to utilize environmental resources [32–35]. *H*. *sosnowskyi* plants of different age stages may compete due to differences in size, growth rhythm, and survival ability during a resource deficit. The structure of the *H*. *sosnowskyi* monostand canopy is maintained due to differences in the physiological traits of plants from different age groups. Seedling leaves require more light energy (IRA = 95 μM photons m^-2^ s^-1^) to begin intensive CO_2_ assimilation than leaves of adult vegetative and generative plants (IRA = 70–72 μM photons m^-2^ s^-1^). This feature of seedling leaves maintains seedlings in “stand-by” mode until the older and higher plants are damaged.

The monostand sustainability is also due to the formation of five or six dormant underground shoot buds in the second and subsequent years of life. Taking into account the density of adult vegetative and generative plants the bank of dormant underground shoot buds is about 20–24 bud per square meter. Disturbances to the aerial portions of the shoots trigger the end of bud dormancy and the start of rapid shoot elongation. The damaged generative plants are then able to form new umbels in a couple of weeks. This underground bud bank allows for the renewal of the aerial plant parts up to six times after damage occurs.

Fast renewal of the monostand structure is also due to the significant and transient soil seed bank (most seeds overcome dormancy within one year). The *H*. *sosnowskyi* soil seed bank can be classified as Type II in terms of the Thompson and Grime classification [36]: the transient seed bank consists of large seeds with the maximum density over the winter period and the ability to germinate at extremely low temperatures. Mass germination and the development of juvenile plants takes place in spring, before plants of older age stages intercept the light. The majority of the juvenile plants drastically slow their development after the formation of the dense canopy by higher plants, but start to grow intensively after gap formation in the canopy, preventing the establishment of other plant species in the monostand.

*H*. *sosnowskyi* plants use environmental resources with relatively high efficiency. Leaves located at different heights are adapted to different light levels, with variation in lamina thickness and photosynthetic pigment content. Upper leaves differ significantly from lower leaves in terms of photosynthetic light-use efficiency and maximum photosynthesis rate. The adaptation irradiance showed that *H*. *sosnowskyi* plants are able to assimilate CO_2_ effectively in conditions of self-shading.

We suggest that early commencement of growth, rapid formation of a dense canopy, high efficiency of light and water use during photosynthesis, ability of young plants to survive under low light conditions, rapid recovery of above-ground plant parts after damage, and the high density of the soil seed bank are the most important factors for the formation of *H*. *sosnowskyi* monostands in invaded areas.
